# Investigation of the Disparity Between New York City and National Prevalence of Nonspecific Psychological Distress Among Hispanics

**DOI:** 10.5888/pcd9.110104

**Published:** 2012-02-09

**Authors:** Sandra S. Albrecht, Katharine H. McVeigh

**Affiliations:** Center for Social Epidemiology and Population Health, University of Michigan. Ms Albrecht is also affiliated with the New York City Epi Scholars Program, New York City Department of Health and Mental Hygiene, New York, New York; Bureau of Epidemiology Services, New York City Department of Health and Mental Hygiene, New York, New York

## Abstract

**Introduction:**

In New York City, the age-adjusted prevalence of nonspecific psychological distress (NPD) among Hispanics is twice that of non-Hispanic whites; nationally, there is little Hispanic-white disparity. We aimed to explain the pattern of disparity in New York City.

**Methods:**

Data came from the 2006 National Health Interview Survey and 2006 Community Health Survey in New York City. Respondents with scores higher than 12 on the K6, a brief scale used to screen for mental health disorders, were defined as having NPD. Multivariate analyses controlled for Hispanic ancestry, socioeconomic status (education, employment, and income), nativity, language of interview, and health characteristics.

**Results:**

In New York City, the disparity between Hispanics and whites was fully explained after accounting for the disproportionate concentration of low socioeconomic status among Hispanics (odds ratio for NPD, 0.81; 95% confidence interval, 0.60-1.11). These factors also partially accounted for differences between Hispanics in New York City and the United States, but the prevalence of NPD overall in New York City remained elevated relative to the United States.

**Conclusion:**

Elevated NPD prevalence among New York City Hispanics was primarily attributable to large disparities in socioeconomic status; differences between New York City and the United States remained but were not specific to Hispanics. Interventions in New York City aimed at addressing racial/ethnic disparities in health may overlap with those addressing socioeconomic inequalities. Further study into the higher overall prevalence of NPD in New York City will be necessary to inform the design and targeting of interventions.

## Introduction

Mental disorders are leading causes of illness and death in the United States (1). Nonspecific psychological distress (NPD) is a group of affective symptoms common to a range of psychiatric disorders but not specific to any single disorder (2). National and community-level surveillance of NPD is necessary for targeting interventions to prevent more serious disease.

Although the prevalence of chronic disease is higher among Hispanics, the largest minority group in the United States, compared with non-Hispanic whites, studies have noted the absence of a similarly patterned disparity in NPD. Many studies, for example, demonstrate similar or lower prevalence of NPD and other adverse mental health outcomes among Hispanics compared with whites (3,4-6). New York City is an exception to this pattern; NPD prevalence is almost twice as high among New York City Hispanics as among non-Hispanic whites (7).

These differences in prevalence remain poorly understood. The differential distribution of known correlates of NPD may provide one explanation. Female sex, low socioeconomic status (SES), marital disruption (divorced, separated, widowed), chronic illness, and Spanish language preference have been well characterized in the literature as risk factors for NPD (3,7-11). The prevalence of these correlates may be higher among Hispanics in New York City relative to non-Hispanic whites in New York City and Hispanics in the United States. Whether this higher prevalence translates to elevated distress is unknown.

Variation in distress and depressive outcomes among ethnicities within the broad "Hispanic" category may also provide an explanation for the disparity between New York City and US Hispanics. Puerto Rican ethnicity, for example, has been associated with a higher risk of adverse mental health relative to Cuban, Mexican, and other Hispanic groups (6,12-15). These differences may arise from social factors associated with discrimination, migration, and culture (16). Most US Hispanics are Mexican; by contrast, the New York City Hispanic population is more heterogeneous, composed predominantly of Puerto Ricans and Dominicans (17). In light of findings that highlight intragroup variation in mental health outcomes among Hispanics, we anticipated that different NPD prevalence estimates between New York City and the United States might be partly attributed to compositional differences in the Hispanic population.

Using nationally and city-representative data for the United States and New York City, we explored how these factors could explain the elevated NPD risk among New York City Hispanics relative to both New York City non-Hispanic whites and US Hispanics. These findings may guide the design and targeting of mental health interventions.

## Methods

### Data sources

We used data from non-Hispanic white and Hispanic participants aged 18 years or older from the 2006 National Health Interview Survey (NHIS) and the 2006 Community Health Survey (CHS) in New York City. NHIS is conducted annually by the National Center for Health Statistics and represents the noninstitutionalized population of the United States. The survey collects detailed information on sociodemographic indicators and mental and physical health, through in-person household interviews conducted in English or Spanish (18). Because NHIS is publicly available and uses de-identified information, analyses using these data were exempt from review by an institutional review board. In 2006, the interviewed adult sample included 24,275 people. Among these, 496 did not respond to questions about distress. After restricting the sample to respondents who self-identified as non-Hispanic white or Hispanic, our US sample consisted of 18,405 adults.

CHS is an annual, random-digit–dialed telephone survey of approximately 10,000 noninstitutionalized New York City adults conducted by the New York City Department of Health and Mental Hygiene. Data collection and analyses were approved by the agency's institutional review board. CHS is designed as a stratified random sample to provide neighborhood and citywide estimates for mental and physical health indicators (19). Data were collected through computer-assisted telephone interviews conducted in multiple languages. Of the 9,683 interviews conducted in 2006, 51 were excluded because of item nonresponse. Restricting this sample to respondents identifying as non-Hispanic white or Hispanic yielded 6,148 adults.

### Dependent variable

NPD was measured using the K6, a brief scale validated for use across racial/ethnic groups that is designed to screen the general population for mental health disorders (20,21). The scale, used by both the 2006 NHIS and the 2006 CHS, was developed using item response theory, a process that selects the best subset of items from a larger universe of items, and is characterized by high specificity and limited differential sensitivity across racial/ethnic groups. To characterize NPD, questions from different mental health surveys were entered into a model and the 6 best questions were selected. The K6 asks respondents how often in the preceding 30 days they felt "sad," "nervous," "restless," "hopeless," "worthless," or that "everything was an effort." Responses are measured on a scale from 0 ("none of the time") to 4 ("all of the time") then summed (range, 0-24). By convention, respondents with K6 scores higher than 12 were classified as having NPD (20).

### Independent variables

Our analyses were limited to the following 6 racial/ethnic groups: non-Hispanic whites, Puerto Ricans, Dominicans, Mexicans, Central/South Americans, and Other Hispanics. Covariates were selected a priori and included those identified in the literature as traditional correlates of distress: age, sex, marital status (married, marital disruption, never married), nativity (United States, other), interview language (English, other), education (less than high school, high school graduate, some college, college graduate), employment status (employed, unemployed, homemaker, retired, student), and poverty-income ratio, which relates a household's annual income to the federal poverty threshold for a household of its size and composition (<100%, 100%-199%, 200%-399%, ≥400%, unknown) (7-10). We also examined health indicators associated with distress in the literature: self-rated health status (excellent/good, fair/poor), diabetes status (ever having it or not), and current asthma (yes, no) (6,7)

### Statistical analysis

All analyses were conducted using SUDAAN version 9 (Research Triangle Institute, Research Triangle Park, North Carolina) with Taylor series linearization methods to adjust for the complex survey design. Recommended weights were applied to both NHIS and CHS data to produce representative NPD prevalence estimates for each racial/ethnic category in the surveys. All estimates were age-adjusted to the 2000 US standard population (22). We conducted bivariate analyses of NPD for selected sociodemographic characteristics, stratified by survey and ethnic group. Comparisons among racial/ethnic groups and other sociodemographic characteristics within each survey were evaluated using the *t* statistic. *P* values of less than .05 were considered significant.

After comparing groups within each survey, we expanded our analyses to compare between surveys. Data from NHIS and CHS were pooled and reweighted to allow for statistical comparisons between the US and New York City populations. Using a series of multivariate logistic regression models, each of which included an interaction between race/ethnicity and survey source (ie, New York City population vs US population), we analyzed the role of known NPD correlates in accounting for the difference between the Hispanic-white gradient in New York City and that in the United States. Our analyses first treated Hispanics as a single group and then separated the various Hispanic ancestry groups. Base models adjusted only for age, and then in a series of richer models sequentially added other demographic characteristics, SES indicators (education, employment, and income), nativity and interview language, and health status measures. We also assessed the interaction of these covariates with survey source because their associations with NPD in New York City differed from their associations in the rest of the United States.

## Results

Puerto Ricans (30.5%), Dominicans (25.9%), and Central/South Americans (22.8%) were the most common groups of New York City Hispanics, whereas in the rest of the United States, most Hispanics were Mexican (61.2%), followed by Central/South American (15.6%). In both New York City and the United States, Hispanics were disproportionately young, had lower SES, and were more likely to self-report fair or poor health (Table 1). These patterns were generally consistent across Hispanic ancestry groups, although high unemployment appeared to be most concentrated among Puerto Ricans and Dominicans in both New York City and the United States. New York City Hispanics had significantly higher rates of marital disruption, low SES, non–English language interviews, and fair or poor health compared with US Hispanics. Many of these differences were concentrated among Puerto Ricans.

The distribution of risk factors differed among ethnicities in New York City compared with the rest of the United States. In New York City, we observed slightly higher, though nonsignificant rates of marital disruption among Hispanics compared with whites; in the United States, this pattern was reversed. The Hispanic-white disparity in SES and health was also considerably wider in New York City.

In New York City, NPD was twice as common among Hispanics (9.3%) as among whites (4.8%) (Figure). Prevalence was highest among Puerto Ricans (11.7%), and Dominicans (9.8%), the 2 largest ancestry groups in New York City, and Other Hispanics (9.9%), and all estimates were significantly higher than those of New York City whites (4.8%). In the United States, among Mexicans, prevalence was only 2.9%, and no Hispanic group showed significantly higher rates of NPD compared with whites (2.8%). Compared with US Hispanics overall (2.7%), New York City Hispanics had 3 times the prevalence of NPD. With the exception of Mexicans, every New York City racial/ethnic group (including whites) had significantly higher NPD estimates compared with its respective US racial/ethnic counterpart.

**Figure.**
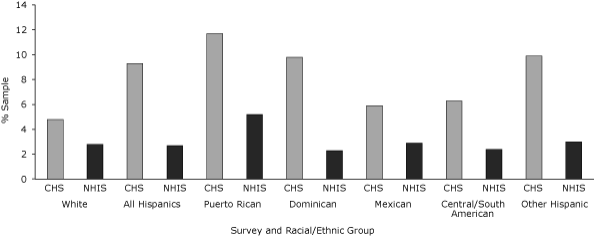
Age-adjusted prevalence of nonspecific psychological distress by race/ethnicity, Community Health Survey (CHS) 2006 and National Health Interview Survey (NHIS) 2006. Within CHS, the difference between the white reference group and all Hispanic groups except Mexicans and Central/South Americans was significant at *P* < .05. Within NHIS, there were no significant differences across racial/ethnic groups. The difference between CHS and NHIS for all racial/ethnic groups except Mexican was significant at *P* < .05. Nonspecific psychological distress defined as a score of >12 on the K6 scale.

**Table:** 

**Racial/Ethnic Group**	**CHS, %**	**NHIS, %**
Whites	4.8	2.8
All Hispanics	9.3	2.7
Puerto Ricans	11.7	5.2
Dominicans	9.8	2.3
Mexicans	5.9	2.9
Central/South Americans	6.3	2.4
Other Hispanic	9.9	3

### Bivariate analyses

In most cases, NPD was especially prevalent among the middle-aged, women, and people of lower SES (Table 2). A notable exception was among US Hispanics, where no education gradient was observed. For several risk factors, the association with NPD was more nuanced and likely to be context-specific. For example, prevalence was higher among non–English-speaking respondents in New York City, but not in the United States. For covariates associated with NPD, New York City Hispanics had a similar or higher prevalence than New York City whites, whereas in the United States, prevalence was higher among whites than Hispanics. At almost every level of each of the covariates examined, New York City Hispanics also had significantly higher NPD compared with US Hispanics.

### Multivariate analyses

In Model 1, New York City Hispanics overall had twice the age-adjusted odds of NPD as New York City whites (Table 3). Both US whites and US Hispanics had similar odds of NPD — about half those of New York City whites. Taken together, these results demonstrate a large age-adjusted gap between whites and Hispanics in New York City and none in the United States. The interaction between race/ethnicity and survey source indicates not only that the Hispanic-white disparity was significantly larger in New York City compared with the United States, but that the difference between the gaps was also significant (*P* < .001).

When Model 1 was used to assess potential differences by Hispanic ancestry, underlying context-specificity in the Hispanic-white disparity became apparent. The results indicate that differences in the distribution of Hispanic ancestry groups between New York City and the United States are not sufficient to explain this context specificity; the disparity between each ancestry group and whites was significantly larger (*P* = .02) in New York City compared with the analogous gap in the United States.

In subsequent models, we accounted for an increasing number of risk factors. The overall gaps between New York City and the United States were robust; that is, accounting for these risk factors did not explain why NPD was more prevalent in New York City. However, these characteristics do explain the ethnicity gap in New York City. Comparing Model 3 to the previous 2 models, the disproportionate socioeconomic disadvantage of New York City Hispanics (and among Puerto Ricans, Dominicans, and Other Hispanics) compared with whites fully accounted for the higher odds of NPD. In the United States, racial/ethnic differences that had been absent emerged: Hispanics overall, and specifically, Dominicans and Mexicans, had lower odds of NPD than did US whites. The interaction between race/ethnicity and survey, however, was not significant, thus confirming that the contextual differences in the Hispanic-white disparity were fully explained by accounting for SES indicators.

Further adjustment for nativity and language of interview in Model 4 actually reversed the race/ethnicity gap in New York City and preserved it in the United States, although it was no longer significant. In New York City, this finding extended specifically to Dominicans, Mexicans, and Central/South Americans, whereas in the United States, Dominicans maintained this advantage. This regression indicated high NPD odds specific to non–English-speaking interviewees only in New York City (data not shown).

Additional controls for health status indicators (ever having diabetes, current asthma, and self-reported health) did not alter estimates from Model 4 and are therefore not shown.

## Discussion

Our findings indicate that socioeconomic disadvantage is a key explanation for why the prevalence of NPD is so much higher among Hispanics than among whites in New York City, a pattern not found in the broader United States. We show that socioeconomic disparities between Hispanics and whites are larger in New York City than in the United States. Moreover, we provide evidence that NPD risk factors are more strongly correlated with disease in New York City compared with the United States. Accounting for these patterns fully explained the NPD gradient in New York City, whether we treated all Hispanics as a single group or categorized them by ancestry. After further accounting for nativity and language of interview, the disparity in New York City reversed and became consistent with that observed in the United States. In New York City, this pattern was observed most strongly among Dominicans, Mexicans, and Central/South Americans.

Our finding that NPD prevalence varies among Hispanics is consistent with the established literature (4,9). Previous studies have identified Puerto Ricans to be at especially high risk of NPD and other adverse mental health outcomes compared with other Hispanic subgroups, and Mexicans are among the subgroups with the lowest prevalence (6,12,23,24). The patterns we report are consistent with these studies. Previous research has suggested that Mexicans may be protected against the development of mental disorders despite socioeconomic disadvantage, and there is evidence that this protection may not extend to other Hispanic groups (6,25,26). These dynamics suggested that heterogeneity among Hispanics in New York City would have been a plausible explanation for its Hispanic-white disparity in NPD. We confirmed that in New York City, most Hispanics are of Puerto Rican ancestry, whereas in the broader United States they are of Mexican ancestry. However, in our empirical analysis we found that accounting for ancestry differences alone was insufficient to explain the disparity in New York City; further accounting for sociodemographic factors was still necessary.

Although we were able to explain the Hispanic-white disparity in New York City, our analysis could not account for the higher overall prevalence of NPD in New York City compared with the United States. Several factors may explain this result. Differences in survey administration may have played a role. CHS is a landline telephone survey, whereas NHIS is conducted in person; telephone surveys may allow respondents to feel less inhibited about expressing negative feelings. Dynamics of sample-selective attrition from the 2 types of surveys may also be different (27). Beyond these measurement dynamics, contextual factors may also be involved. A higher prevalence of psychiatric disorders is found in urban areas (28,29). Thus, factors associated with residence in New York City, including pace of life, cost of living, and heightened perception of and exposure to terrorism, may play a role in higher NPD prevalence. Future studies in other large metropolitan areas in the United States may shed light on this remaining unanswered question.

Our analyses had additional limitations. First, all data were self-reported; they may conflate cultural norms about affective expression with real psychological impairment. Many studies have documented that less acculturated Hispanic immigrants tend to express more idioms associated with mood and anxiety, known as "nervios." This pattern is observed particularly strongly among Puerto Ricans. Expression of these idioms may not necessarily indicate psychological distress as it is clinically understood (30). Validation of the K6 scale for use across racial/ethnic groups was conducted in nationally representative samples (20). National samples of Hispanics consist mostly of Mexicans; validity of the scale may not extend to other Hispanic subgroups such Puerto Ricans and Dominicans. We also noted a higher NPD prevalence among non–English language respondents in New York City, including non-Hispanic whites. This finding may warrant deeper examination of other non–English language versions of the K6 scale.

Despite these limitations, we capitalized on the availability of large samples representing the populations of New York City and the United States. We accounted for differences in the ethnic composition of Hispanics as a possible factor influencing distress rates. Our analyses point to the central role of the Hispanic-white socioeconomic gradient in New York City in explaining the city's racial/ethnic disparity in NPD. Our findings suggest that mental health interventions should be targeted to low-SES populations and should address socioeconomic risk factors directly. Future research is warranted to investigate the reasons underlying the elevated overall prevalence of NPD in New York City to inform design and targeting of interventions citywide and in other urban populations.

## Figures and Tables

**Table 1.** Sample Characteristics by Race/Ethnicity, CHS 2006 and NHIS 2006

**Characteristic**	New York City Race/Ethnicity (CHS), %						

	Whites (n = 3,765)^a^	All Hispanics (n = 2,383)^a^	Puerto Rican (n = 831)^a^	Dominican (n = 597)^a^	Mexican (n = 200)^a^	Central/South American (n = 514)^a^	Other Hispanic (n = 241)^a^
Age ≥65 y	22.5^b^	9.4^c^	14.5^c^	8.5^c^	1.6^b^^,^ c	7.7^c^	12.0^c^
Female	51.6	55.1^b^	60.5^b^^,^ c	57.5	47.7	52.2	49.1
Marital disruption^d^	19.0	21.3^b^	25.5^b^^,^ c	23.9^c^	11.1^c^	19.2	21.4
<High school education	6.6^b^	36.0^b^^,^ c	30.4^c^	40.3^c^	56.0^b^^,^ c	29.8^c^	27.5^b^^,^ c
Unemployed	7.9	17.4^b^^,^ c	24.9^b^^,^ c	20.7^c^	11.4	10.1^b^	12.0
Poverty-income ratio^e^ <100%	8.9^b^	31.9^b^^,^ c	25.7^b^^,^ c	33.9^c^	54.6^b^^,^ c	28.6^b^^,^ c	21.3^b^^,^ c
Foreign-born	24.8^b^	58.1^c^	1.3^b^^,^ c	82.1^c^	93.4^b^^,^ c	83.9^c^	53^b^^,^ c
Non-English interview language	6.6^b^	47.5^b^^,^ c	18.4^b^^,^ c	63.7^b^^,^ c	83.1^b^^,^ c	52.9^b^^,^ c	27.5^c^
Fair/poor self-reported health	16.7^b^	26.2^b^^,^ c	29.5^b^^,^ c	31.1^b^^,^ c	20.7^b^	20.9^b^	23.8^b^^,^ c

**Characteristic**	**US Race/Ethnicity (NHIS), %**						

	**Whites (n = 14,243)^a^**	**All Hispanics (n = 4,162)^a^**	**Puerto Rican (n = 422)^a^**	**Dominican (n = 138)^a^**	**Mexican (n = 2,581)^a^**	**Central/South American (n = 647)^a^**	**Other Hispanic (n = 374)^a^**

Age ≥65 y	18.6	8.1^c^	13.3^c^	6.9^c^	6.4^c^	7.7^c^	16.5
Female	51.8	48.7^c^	51.8	63.9^c^	46.9^c^	51.2	48.8
Marital disruption^d^	17.5	13.1^c^	17.7	22.8	11.0^c^	15.9	15.0
<High school education	12.1	40.2^c^	30.1^c^	39.5^c^	46^c^	35.8^c^	17.9^c^
Unemployed	8.5	9.4	15.4^c^	21.3^c^	8.8	6.3	9.6
Poverty-income ratio^e^ <100%	6.6	16.8^c^	18.2^c^	26.0^c^	17.3^c^	16.2^c^	10.5^c^
Foreign-born	4.4	61.1^c^	50.7^c^	82.1^c^	57.5^c^	88.5^c^	42.7^c^
Non-English interview language	0.3	26.4^c^	9.8^c^	28.6^c^	27.1^c^	34.3^c^	24.8^c^
Fair/poor self-reported health	11.1	13.6^c^	15.7^c^	15.5^c^	13.5^c^	11.5	15.1^c^

Abbreviations: CHS, Community Health Survey; NHIS, National Health Interview Survey.

a Unweighted sample size.

b *P* < .05, comparing each New York City racial/ethnic group to its respective US counterpart.

c *P* < .05, comparing each Hispanic group to whites within survey.

d Divorced, separated, or widowed.

e Ratio of household income from all sources to the federal poverty threshold for same year.

**Table 2.** Age-Adjusted Prevalence of Nonspecific Psychological Distress^a^, by Selected Characteristics, CHS 2006 and NHIS 2006

**Characteristic**	New York City (CHS), %		United States (NHIS), %		Difference^b^	

	Whites (n = 3,765)	Hispanics (n = 2,383)	Whites (n = 14,243)	Hispanics (n = 4,162)	Whites	Hispanics
**Age, y**						
18-24	2.1	4.6	2.0	1.6	0.1	3.0
25-44	3.3	7.3^c^	2.7	2.1	0.6	5.2^d^
45-64	6.6	14.2^c^	3.9	4.6	2.7	9.6^d^
≥65	7.4	8.9	2.0	3.0	5.4	5.9^d^
**Sex**						
Male	3.9	6.9^c^	2.3	1.8	1.6^d^	5.1^d^
Female	5.6	11.2^c^	3.4	4.1	2.2^d^	7.1 d
**Marital status**						
Married/cohabitating	3.0	6.3^c^	2.4	2.5	0.6	3.8^d^
Marital disruption^e^	11.8	11.8	5.5	4.5	6.3^d^	7.3^d^
Never married	5.1	12.6^c^	4.1	3.0	1.0	9.6^d^
**Education**						
<High school	10.9	13.6	7.7	2.7^c^	3.2	10.9^d^
High school	7.5	7.5	2.8	3.2	4.7^d^	4.3^d^
Some college	4.6	6.7	3.0	2.6	1.6	4.1^d^
College graduate	3.3	3.7	1.0	3.4	2.3^d^	0.3
**Employment**						
Employed	2.8	7.1^c^	1.3	1.2	1.5	5.9^d^
Unemployed	20.2	18.8	16.3	10.6^c^	3.9	8.2^d^
Homemaker	3.2	9.1^c^	2.7	2.2	0.5	6.9^d^
Retired	6.6	2.4	0.7	3.5	5.9	−1.1
Student	1.0	21.9^c^	2.3	3.5	−1.3	18.4
**Poverty-income ratio, %^f^**						
<100	12.6	13.1	9.3	5.8^c^	3.3	7.3^d^
100-199	10.8	10.2	7.7	1.9^c^	3.1	8.3^d^
200-399	8.0	6.8	2.1	3.1	5.9^d^	3.7
≥400	1.9	2.0	1.3	2.5	0.6	−0.5
**Nativity**						
US-born	4.1	10.4^c^	2.8	3.3	1.3^d^	7.1^d^
Foreign-born	6.7	8.5	3.1	2.8	3.6^d^	5.7^d^
**Language of interview**						
English	4.0	8.0^c^	2.8	3.1	1.2^d^	4.9^d^
Other language	13.2	10.4	3.2	2.5	10.2^d^	7.9^d^
**Ever had diabetes**						
Yes	11.0	25.7^c^	8.3	5.0	2.7	20.7^d^
No	4.4	8.3^c^	2.5	2.6	1.9^d^	5.7^d^
**Current asthma**						
Yes	17.7	25.4	6.5	8.1	11.2^d^	17.3^d^
No	4.2	8^c^	2.5	2.7	1.7^d^	5.3^d^
**Self-rated health**						
Good or excellent	2.4	4.6^c^	1.5	1.6	0.9^d^	3^d^
Fair or poor	17.6	20.1	16.4	9.4^c^	1.2	10.7^d^

Abbreviations: CHS, Community Health Survey; NHIS, National Interview Survey.

a Refers to a group of affective symptoms common to a range of psychiatric disorders but not specific to any single disorder (2). Defined for this study as a score of >12 on the K6 scale (20).

b Difference between New York City Hispanics vs US Hispanics and New York City whites vs US whites.

c *P* < .05, comparing difference between Hispanics and whites within survey for each sample characteristic.

d *P* < .05, comparing New York City Hispanics and whites each to their respective US ethnic counterparts.

e Divorced, separated, or widowed.

f Ratio of household income from all sources to the federal poverty threshold for same year.

**Table 3.** Adjusted Odds of Nonspecific Psychological Distress^a^, by Race/Ethnicity, New York City vs the United States, CHS 2006 and NHIS 2006

**Race/Ethnicity Panel**	Model 1 (Age-Adjusted)^b^	Model 2 (Adds Sex and Marital Status)	Model 3 (Adds SES)^c^	Model 4 (Adds Nativity and Interview Language)^d^
**All Hispanic groups combined**				
New York City Hispanics (reference, New York City whites), AOR (95% CI)	2.09 (1.65-2.64)	1.87 (1.47-2.37)	0.81 (0.60-1.11)	0.63 (0.45-0.90)
US whites (reference, New York City whites), AOR (95% CI)	0.56 (0.43-0.72)	0.59 (0.46-0.77)	0.46 (0.34-0.62)	0.50 (0.37-0.67)
US Hispanics (reference, New York City whites), AOR (95% CI)	0.55 (0.39-0.77)	0.58 (0.41-0.83)	0.32 (0.21-0.48)	0.37 (0.22-0.60)
US Hispanics (reference, US whites), AOR (95% CI)	0.98 (0.75-1.28)	0.99 (0.76-1.29)	0.69 (0.50-0.95)	0.74 (0.48-1.13)
**Interaction of race/ethnicity and survey, *P* value**	<.001	<.001	.40	.58
**Hispanic groups separated by ancestry (reference, non-Hispanic white in same survey)**				
**Puerto Ricans, AOR (95% CI)**				
New York City	2.58 (1.90-3.49)	2.14 (1.57-2.90)	0.79 (0.53-1.16)	0.71 (0.46-1.10)
United States	1.80 (1.02-3.17)	1.71 (0.97-3.02)	0.92 (0.49-1.74)	0.94 (0.49-1.79)
**Dominicans, AOR (95% CI)**				
New York City	2.26 (1.58-3.25)	1.99 (1.37-2.88)	0.80 (0.51-1.25)	0.50 (0.30-0.85)
United States	0.69 (0.34-1.39)	0.59 (0.30-1.14)	0.26 (0.12-0.54)	0.27 (0.12-0.64)
**Mexicans, AOR (95% CI)**				
New York City	1.63 (0.86-3.09)	1.74 (0.91-3.29)	0.67 (0.30-1.48)	0.37 (0.15-0.88)
United States	0.90 (0.66-1.23)	0.94 (0.68-1.28)	0.64 (0.44-0.94)	0.68 (0.41-1.10)
**Central/South Americans, AOR (95% CI)**				
New York City	1.37 (0.89-2.11)	1.29 (0.84-2.01)	0.78 (0.47-1.29)	0.51 (0.29-0.89)
United States	0.83 (0.41-1.70)	0.79 (0.38-1.62)	0.71 (0.33-1.51)	0.74 (0.34-1.64)
**Other Hispanics, AOR (95% CI)**				
New York City	2.24 (1.24-4.04)	2.10 (1.14-3.90)	1.25 (0.57-2.74)	1.01 (0.46-2.22)
United States	1.06 (0.41-2.74)	1.09 (0.42-2.82)	0.94 (0.31-2.85)	0.99 (0.32-3.10)
**Interaction of race/ethnicity and survey, *P* value**	.02	.02	.20	0.36

Abbreviations: CHS, Community Health Survey (New York City); NHIS, National Interview Survey; SES, socioeconomic status; AOR, adjusted odds ratio; CI, confidence interval.

a Refers to a group of affective symptoms common to a range of psychiatric disorders but not specific to any single disorder (2). Defined for this study as a score of >12 on the K6 scale (20).

b Includes interaction between age (reference group, age 45-64 y) and survey.

c SES indicators were education, income, and employment status.

d Includes interaction between interview language (reference, English) and survey.

## References

[1] Croft JB, Mokdad AH, Power AK, Greenlund KJ, Giles WH (2009). Public health surveillance of serious psychological distress in the United States. Int J Public Health.

[2] Dohrenwend BP, Shrout PE, Egri G, Mendelsohn FS (1980). Nonspecific psychological distress and other dimensions of psychopathology. Measures for use in the general population. Arch Gen Psychiatry.

[3] Alegria M, Canino G, Shrout PE, Woo M, Duan N, Vila D (2008). Prevalence of mental illness in immigrant and non-immigrant US Latino groups. Am J Psychiatry.

[4] Breslau J, Aguilar-Gaxiola S, Kendler KS, Su M, Williams D, Kessler RC (2006). Specifying race-ethnic differences in risk for psychiatric disorder in a USA national sample. Psychol Med.

[5] Breslau J, Kendler KS, Su M, Gaxiola-Aguilar S, Kessler RC (2005). Lifetime risk and persistence of psychiatric disorders across ethnic groups in the United States. Psychol Med.

[6] Bratter JL, Eschbach K (2005). Race/ethnic differences in nonspecific psychological distress: evidence from the National Health Interview Survey. Soc Sci Q.

[7] McVeigh KH, Galea S, Thorpe LE, Maulsby C, Henning K, Sederer LI (2006). The epidemiology of nonspecific psychological distress in New York City, 2002 and 2003. J Urban Health.

[8] Vega WA, Rumbaut RG (1991). Ethnic minorities and mental health. Annu Rev Sociol.

[9] Strine TW, Dhingra SS, Okoro CA, Zack MM, Balluz LS, Berry JT (2009). State-based differences in the prevalence and characteristics of untreated persons with serious psychological distress. Int J Public Health.

[10] Kessler RC, Berglund P, Demler O, Jin R, Koretz D, Merikangas KR (2003). The epidemiology of major depressive disorder: results from the National Comorbidity Survey Replication (NCS-R). JAMA.

[11] Alegria M, Canino G, Stinson FS, Grant BF (2006). Nativity and DSM-IV psychiatric disorders among Puerto Ricans, Cuban Americans, and non-Latino whites in the United States: results from the National Epidemiologic Survey on Alcohol and Related Conditions. J Clin Psychiatry.

[12] Moscicki EK, Rae DS, Regier DA, Locke BZ, Gaviria M, Arana JD (1987). The Hispanic Health and Nutrition Examination Survey: depression among Mexican Americans, Cuban Americans and Puerto Ricans. Health and behavior: research agenda for Hispanics.

[13] Potter LB, Rogler LH, Moscicki EK (1995). Depression among Puerto Ricans in New York City: the Hispanic Health and Nutrition Examination Survey. Soc Psychiatry Psychiatr Epidemiol.

[14] Yang FM, Cazorla-Lancaster Y, Jones RN (2008). Within-group differences in depression among older Hispanics living in the United States. J Gerontol B Psychol Sci Soc Sci.

[15] Guarnaccia PJ, Good BJ, Kleinman A (1990). A critical review of epidemiological studies of Puerto Rican mental health. Am J Psychiatry.

[16] Zsembik BA, Fennell D (2005). Ethnic variation in health and the determinants of health among Latinos. Soc Sci Med.

[17] (2001). Census 2000 brief: the Hispanic population 2000.

[18] (2007). 2006 National Health Interview Survey (NHIS) public use release.

[19] Community Health Survey 2006 methods.

[20] Kessler RC, Andrews G, Colpe LJ, Hiripi E, Mroczek DK, Normand SL (2002). Short screening scales to monitor population prevalences and trends in non-specific psychological distress. Psychol Med.

[21] Kessler RC, Barker PR, Colpe LJ, Epstein JF, Gfroerer JC, Hiripi E (2003). Screening for serious mental illness in the general population. Arch Gen Psychiatry.

[22] Klein RJ, Schoenborn CA (2001). Age adjustment using the 2000 projected US population. Healthy People Statistical Notes, no. 20.

[23] Alegria M, Mulvaney-Day N, Torres M, Polo A, Cao Z, Canino G (2007). Prevalence of psychiatric disorders across Latino subgroups in the United States. Am J Public Health.

[24] Shrout PE, Canino GJ, Bird HR, Rubio-Stipec M, Bravo M, Burnam MA (1992). Mental health status among Puerto Ricans, Mexican Americans, and non-Hispanic whites. Am J Community Psychol.

[25] Almeida J, Subramanian SV, Kawachi I, Molnar BE (2009). Is blood thicker than water? Social support, depression and the modifying role of ethnicity/nativity status. J Epidemiol Community Health.

[26] Rodriguez N, Mira CB, Paez ND, Myers HF (2007). Exploring the complexities of familism and acculturation: central constructs for people of Mexican origin. Am J Community Psychol.

[27] Blumberg SJ, Luke JV (2008). Wireless substitution: early release of estimates from the National Health Interview Survey, July-December 2007.

[28] Dhingra SS, Strine TW, Holt JB, Berry JT, Mokdad AH (2009). Rural-urban variations in psychological distress: findings from the Behavioral Risk Factor Surveillance System, 2007. Int J Public Health.

[29] Blazer D, George LK, Landerman R, Pennybacker M, Melville ML, Woodbury M (1985). Psychiatric disorders. A rural/urban comparison. Arch Gen Psychiatry.

[30] Guarnaccia PJ, Lewis-Fernandez R, Marano MR (2003). Toward a Puerto Rican popular nosology: nervios and ataque de nervios. Cult Med Psychiatry.

